# Fat Necrosis of the Breast: A Pictorial Review of the Mammographic, Ultrasound, CT, and MRI Findings with Histopathologic Correlation

**DOI:** 10.1155/2015/613139

**Published:** 2015-03-16

**Authors:** William D. Kerridge, Oleksandr N. Kryvenko, Afua Thompson, Biren A. Shah

**Affiliations:** ^1^Department of Radiology and Imaging Sciences, Indiana University School of Medicine, Indianapolis, IN 46202, USA; ^2^Departments of Pathology and Urology, University of Miami Miller School of Medicine, Miami, FL 33136, USA; ^3^Department of Radiology, Meharry Medical College, Nashville, TN 37208, USA; ^4^Department of Radiology, Henry Ford Hospital, Detroit, MI 48202, USA

## Abstract

Fat necrosis of the breast is a challenging diagnosis due to the various appearances on mammography, ultrasound, CT, PET-CT, and MRI. Although mammography is more specific, ultrasound is a very important tool in making the diagnosis of fat necrosis. MRI has a wide spectrum of findings for fat necrosis and the appearance is the result of the amount of the inflammatory reaction, the amount of liquefied fat, and the degree of fibrosis. While CT and PET-CT are not first line imaging examinations for the diagnosis of breast cancer or fat necrosis, they are frequently performed in the surveillance and staging of disease. Knowledge of how fat necrosis presents on these additional imaging techniques is important to prevent misinterpretation of the imaging findings. Gross and microscopic appearances of fat necrosis depend on the age of the lesion; the histologic examination of fat necrosis is usually straightforward. Knowledge of the variable appearances of fat necrosis on a vast array of imaging modalities will enhance a radiologist's accuracy in the analysis and interpretation of fat necrosis versus other diagnoses.

## 1. Introduction

Fat necrosis is a benign nonsuppurative inflammatory process of adipose tissue. It is important to diagnose fat necrosis because it can often mimic carcinoma of the breast. Fat necrosis in the breast is a common pathologic condition with a wide variety of presentations on mammography, ultrasound, and MRI.

The incidence of fat necrosis of the breast is estimated to be 0.6% in the breast, representing 2.75% of all breast lesions. Fat necrosis is found to be 0.8% of breast tumors and 1% in breast reduction mammoplasty cases. The average age of patients is 50 years [1].

Fat necrosis is most commonly the result of trauma to the breast (21–70%), radiotherapy, anticoagulation (warfarin), cyst aspiration, biopsy, lumpectomy, reduction mammoplasty, implant removal, breast reconstruction with tissue transfer, duct ectasia, and breast infection. Other rare causes for fat necrosis include polyarteritis nodosa, Weber-Christian disease, and granulomatous angiopanniculitis. In some patients, the cause for fat necrosis is unknown [1].

The typical clinical presentation of fat necrosis can range from an incidental benign finding to a lump. However, in around half of the cases patients do not report any injury to the breast and are clinically occult. Following injury to breast tissue, hemorrhage in the fat leads to induration and firmness, which demarcates and may result in a cavity caused by cystic degeneration. The clinical features of fat necrosis vary from indolent single or multiple smooth round nodules to clinically worrisome fixed, irregular masses with overlying skin retraction [2–6]. Other clinical features associated with fat necrosis include ecchymosis, erythema, inflammation, pain, skin retraction or thickening, nipple retraction, and occasionally lymphadenopathy [1, 2]. There is little difference in the clinical presentations of fat necrosis regardless of whether they are related to trauma. In cases related to trauma, the majority of patients presented with a breast lump. The mean time of patients to present with a breast lump from time of trauma is 68.5 weeks. Fat necrosis is commonly seen in the superficial breast tissues and subareolar regions in obese women with pendulous breasts [1]. The aim of this paper is to review the histopathological and radiological features of fat necrosis of the breast which distinguishes it from a cancer.

## 2. Histopathologic Findings of Fat Necrosis

**Figure 1:**
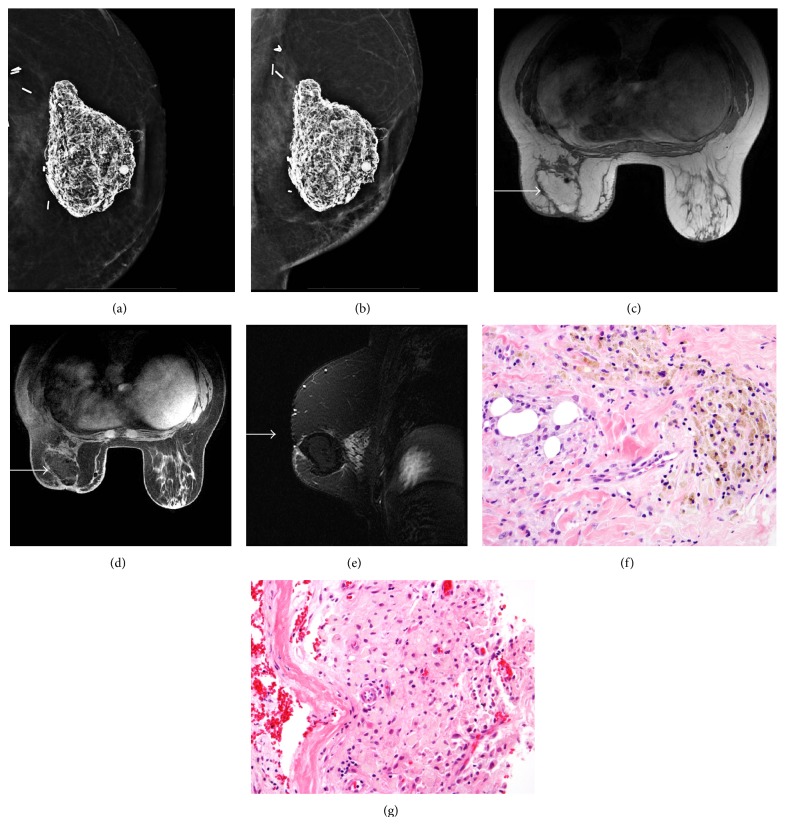
49-year-old female with history of left modified radical mastectomy with transverse rectus abdominis myocutaneous (TRAM) flap reconstruction. Left TRAM flap reconstruction craniocaudal and mediolateral oblique projections ((a) and (b)) demonstrate a large mass of dystrophic calcification and fat. MRI breast T1-weighted nonfat saturation (c), T1-weighted fat saturation after gadolinium (d), and T2-weighted fat saturation images (e) demonstrate a mass in the central left TRAM which follows fat signal on all pulse sequences with a thin rim of enhancement (arrow). The biopsy ((f); H&E, 400x) demonstrating dense fibrotic tissue with foamy histiocytes (left) and hemosiderin-laden macrophages (upper and right). Rebiopsy ((g); H&E, 400x) demonstrating fibrous paucicellular cyst wall (left) surrounded by massive accumulation of foamy histiocytes (center and right).

**Figure 2:**
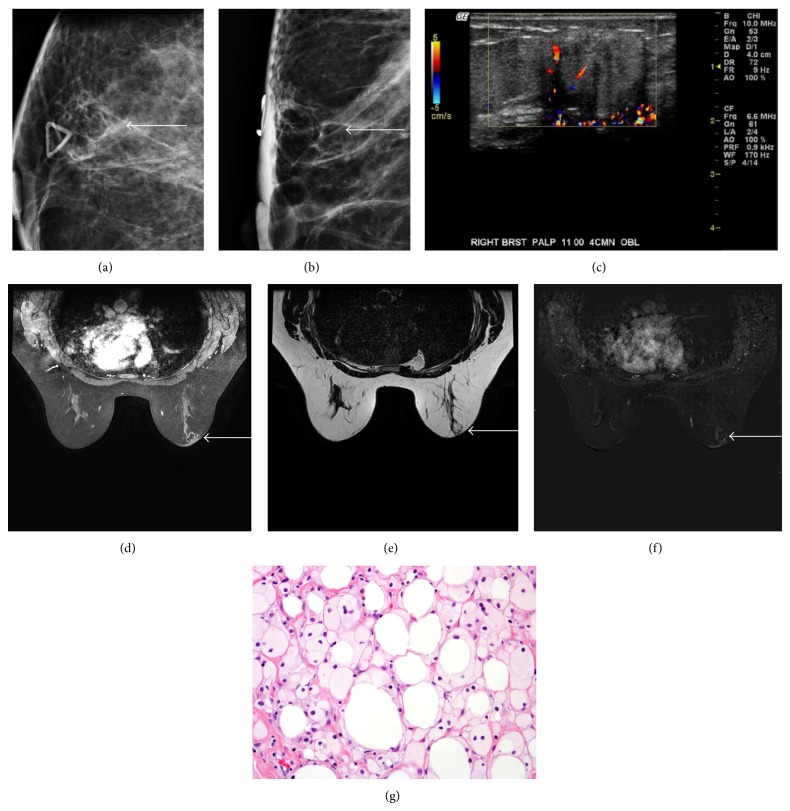
51-year-old female with history of right lobular carcinoma in situ status after lumpectomy and radiation now with palpable lump. Right breast mediolateral oblique and craniocaudal projections ((a) and (b)) demonstrate a radiolucent lobular mass at site of palpable mass (arrow). Targeted ultrasound (c) at site of palpable mass shows a lobular heterogeneous hypoechoic mass with posterior acoustic shadowing. Axial T1-weighted fat saturation after gadolinium, T2-weighted nonfat saturation, and subtraction images ((d)–(f)) demonstrate a mass at 11 o'clock in the right breast anteriorly that follows fat signal on all sequences with thin rim enhancement (arrow). Histologically, tissue specimen ((g); H&E, 400x) demonstrating dead, anucleated adipocytes intermixed with foamy histiocytes.

**Figure 3:**
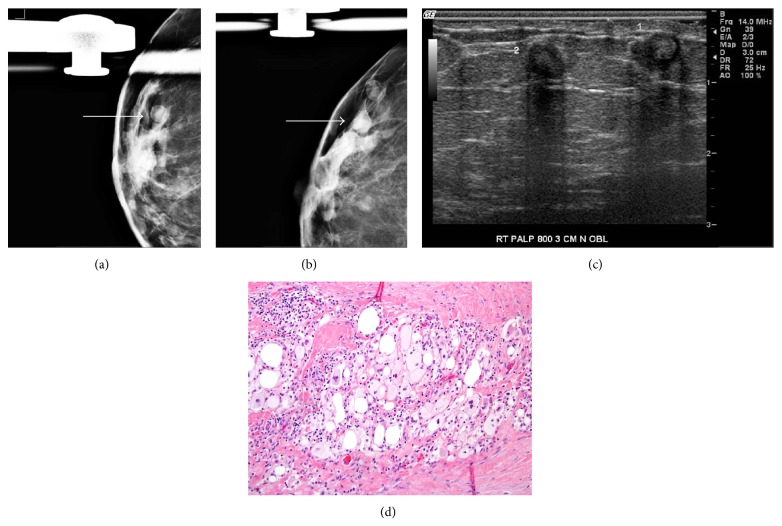
78-year-old female with palpable right breast masses. Right breast mediolateral oblique and craniocaudal mammograms ((a) and (b)) demonstrate round masses with radiolucent centers at the site of palpable finding (arrow). Ultrasound of the right breast (c) at site of palpable finding demonstrate two hypoechoic round masses with central echogenicity with associated posterior acoustic shadowing. Hematoxylin and eosin (H&E, 200x) slide (d) shows fibrous areas around excision cavity with mixed chronic inflammatory cells, foamy histiocytes, and occasional giant cells.

**Figure 4:**
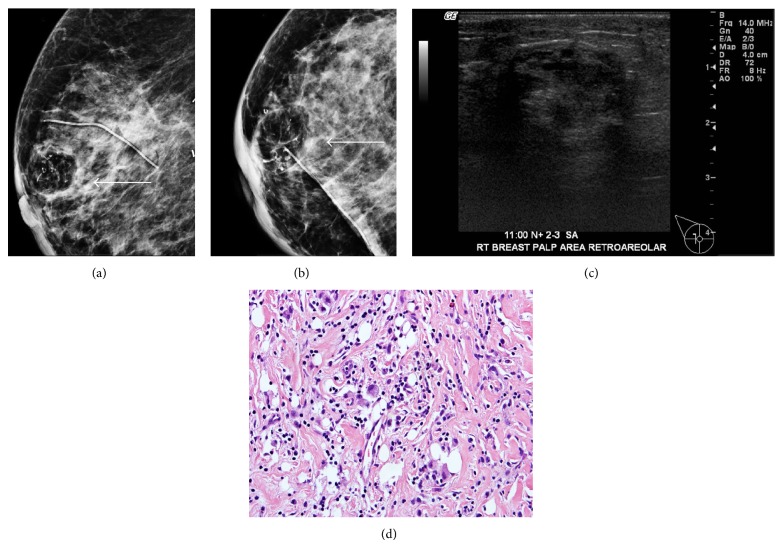
85-year-old female with history of right breast mucinous carcinoma, lobular carcinoma in situ (LCIS), and ductal carcinoma in situ (DCIS) status after lumpectomy and radiation. Right breast mammogram ((a) and (b)) craniocaudal and mediolateral oblique views demonstrate a radiolucent round mass with dystrophic calcifications (arrow) (c). Targeted ultrasound demonstrates a heterogeneous hypoechoic mass with areas of posterior acoustic shadowing. The biopsy ((d); H&E, 400x) demonstrating dense fibrotic tissue with mixed chronic inflammatory infiltrate and scattered foamy histiocytes.

Gross and microscopic appearances of the fat necrosis depend on the age of the lesion. Macroscopically, early lesions appear as hemorrhagic foci or areas of indurated fat. In time, the lesion may become bright yellow (saponification), chalky white (calcification), or yellow-gray (fibrosis). Some lesions may develop a central cavity because of liquefactive necrosis (Figure 1). Poppiti Jr. et al. referred to such cystic lesions as membranous fat necrosis [7]. Microscopically, early lesions show hemorrhage, anucleated adipocytes, foamy (lipid-laden) histiocytes, and multinucleated giant cells (Figure 2). Older lesions develop fibrosis with a few foamy histiocytes and multinucleated giant cells (Figures 3 and 4). However, the latter are usually seen even in older foci of fat necrosis which underwent subsequent transformation. Hemosiderin-laden macrophages may be seen as a morphologic evidence of remote hemorrhage (Figure 1). Dystrophic calcification may occur in older lesions. The morphologic examination of fat necrosis is usually straightforward. If needed, the histogenesis of foamy histiocytes may be confirmed by positive CD68 and negative pan cytokeratin immunostains. However, older lesions with prominent fibrosis may warrant a more scrutinized examination of the specimen and pan cytokeratin immunostaining to rule out invasive lobular carcinoma in which discohesive single cells with small monomorphic nuclei infiltrate the stroma [8].

## 3. Imaging Findings of Fat Necrosis

**Figure 5:**
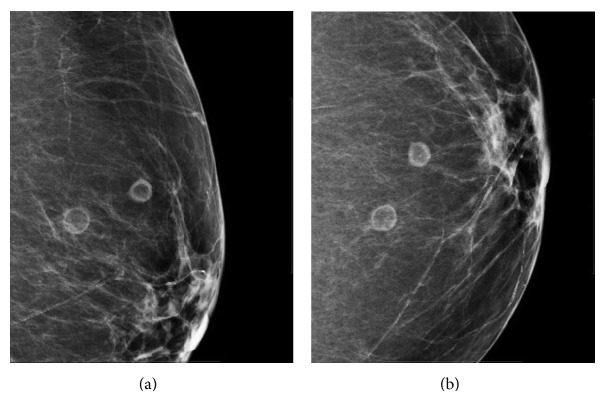
69-year-old asymptomatic female with a stable screening mammogram for 18 years. Left breast mammogram craniocaudal and mediolateral oblique projections demonstrate two round masses with radiolucent centers at 12 o'clock position anteriorly.

On mammography, common findings of fat necrosis are oil cysts (Figure 5), coarse calcifications, focal asymmetries, microcalcifications, or spiculated masses. Lipid cysts are pathognomonic of benign fat necrosis, although the fibrous rim of the cyst may calcify or collapse and may produce an appearance that is mammographically indeterminate and requires biopsy to exclude malignancy (Figure 3). Calcifications may form in the cyst walls and are frequently seen on mammography, usually as smooth and round or curvilinear. Calcifications may be the only findings but may be of concern if they are branching, rod-like, or angular [9]. The clustered, pleomorphic microcalcifications may be indistinguishable from those of malignancy (Figure 4) [10, 11]. When fibrosis is present but the radiolucent fat is not completely replaced, the oil cyst may have thickened, irregular, spiculated, or ill-defined walls. Fibrosis may lead to replacement of the radiolucent necrotic fat, resulting in the appearance of a focal asymmetric density, a focal dense mass, or an irregular spiculated mass on mammography [12]. Oil cysts with fat-fluid levels or serous-hemorrhagic contents, collapsed cysts, and cysts containing spherical densities are all atypical features of fat necrosis.

On sonography, the appearance of fat necrosis ranges from a solid hypoechoic mass with posterior acoustic shadowing to complex intracystic masses that evolve over time. These features depict the histological evolution of fat necrosis. Fat necrosis may appear as cystic or solid masses. Cystic lesions appear complex with mural nodules or internal echogenic bands. Solid masses have circumscribed or ill-defined margins and are often associated with distortion of the breast parenchyma [13]. In a retrospective study of the clinical, mammographic, and sonographic features of fat necrosis by Bilgen et al., 26.9% of lesions demonstrated increased echogenicity of the subcutaneous tissue with or without small cysts, 16.6% were anechoic masses with posterior acoustic enhancement, 14.2% were solid-appearing masses, 11.1% had cystic masses with internal echoes, and 3.9% had cystic masses with mural nodules [14]. A specific sonographic indicator of fat necrosis is a mass with echogenic internal bands that shift in orientation with changes in patient position [15]. Hyperechoic masses very rarely represent malignancy; in fact, hyperechoic cancers are reported in less than 0.8% of tumors [16]. Although rare, malignant hyperechoic lesions include invasive ductal and lobular carcinoma, lymphoma, angiosarcoma, and liposarcoma [16]. The associated ultrasound characteristics (margin, shape, and hypervascularity) are important to consider when determining follow-up or when determining whether core needle biopsy is needed [16].

**Figure 6:**
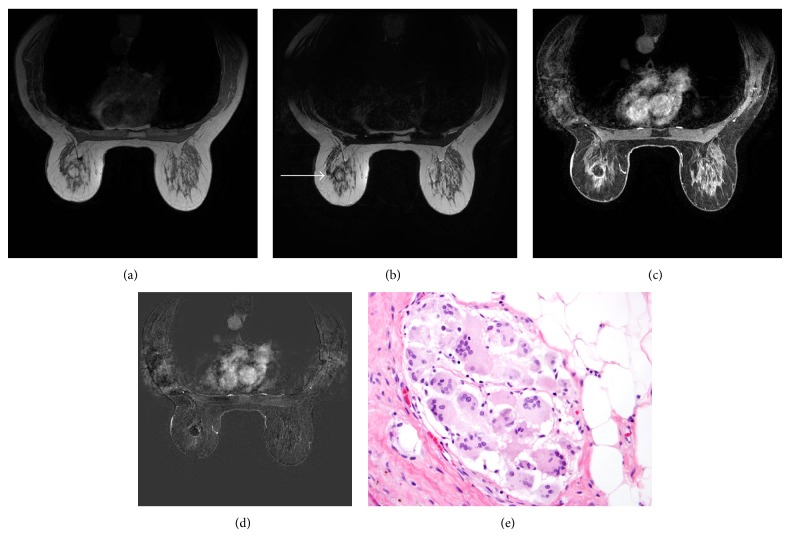
Axial T1-weighted nonfat saturation, T2-weighted nonfat saturation, and T1-weighted fat saturation after gadolinium and subtraction images ((a)–(d)) demonstrate a mass in the left breast which follows fat signal on all sequences (arrow). Histologically, excision ((e); H&E, 400x) shows collection of multinucleated cells in a fibrous area around excision cavity.

MRI also has a wide spectrum of findings for fat necrosis and the appearance is the result of the amount of the inflammatory reaction, the amount of liquefied fat, and the degree of fibrosis. The most common appearance of fat necrosis on MRI is a lipid cyst, round or oval mass with hypointense T1-weighted signal on fat saturation images [17]. Fat necrosis is usually isointense to fat elsewhere in the breast (Figure 2) and shows low signal intensity on T1-weighted MRI, which may be due to its hemorrhagic and inflammatory content [17]. Fat necrosis may show focal or diffuse and homogeneous or heterogeneous enhancement after the administration of IV paramagnetic contrast material. The amount of enhancement is correlated with the intensity of the inflammatory process [17]. As the high signal of fat interferes with the detection of enhancing lesions on MRI, fat suppression is important for identifying enhancing breast cancers or enhancing regions of fat necrosis on MRI [15]. As mentioned before, fat necrosis is usually isointense to fat elsewhere in the breast, a key to diagnosis (Figure 6). In cases where fat necrosis is not isointense, the T1-weighted signal may be lower than fat elsewhere in the breast [12]. Another useful technique for ruling out necrotic tumors is using unenhanced non-fat-saturated T1-weighted images to evaluate the degree of lipid cyst formation, looking for a thin rim of enhancement [17]. Further, the “black hole” sign has been described as another characteristic on MRI to help diagnose fat necrosis, marked central hypointensity of the lesion on short tau inversion recovery (STIR) images when compared with surrounding fat [18]. Fat necrosis may mimic malignancy with varying appearances on MRI. Thin rim of enhancement (Figure 1) is common although it may also be thick, irregular, or spiculated, which are features of recurrent or residual cancer. Another confounding factor in the diagnosis is the different appearance that fat necrosis may have in the same patient. Kinetic analysis may be of little help because fat necrosis exhibits the full spectrum of benign and malignant enhancement patterns. Fat necrosis may also show FDG uptake on PET [19].

CT is not typically included in the imaging protocol for breast cancer detection; however, cancer patients may undergo chest CT as part of staging and surveillance. CT can show areas of fat necrosis and knowledge of the CT appearance will help prevent misinterpretation of the imaging findings. The CT appearance is based on the main components found in fat necrosis: liquefied fat, fibrosis, and inflammation. Liquefied fat would present on CT as low attenuation coefficients, fibrosis would present as soft tissue coefficients similar to fibroglandular tissue or linear densities resembling fibrous bands, and inflammation would present with enhancement after contrast injection [12]. Calcifications typically are not evident until later in the evolution of fat necrosis when they become large in size.

Although F^18^-FDG PET/CT is not recommended for the primary detection of breast cancer, it may play a role in the detection of local recurrence or distant metastases in the setting of locally advanced breast cancer when other imaging modalities are equivocal or confounding [20]. There are several entities within the breast that will show increased FDG-activity on PET/CT with F^18^-FDG. These include acute and chronic inflammation, physiologic lactation, and benign focal breast masses including fat necrosis amongst others. Fat necrosis has increased FDG uptake secondary to the presence of metabolically active inflammatory cells in early stages of the process [19]. Fat necrosis of the breast is often hypermetabolic on PET/CT and may show intense FDG activity in the setting of transverse rectus abdominis myocutaneous (TRAM) flap reconstruction if the fat-rich tissue is damaged intraoperatively [21]. The presentation of a patient with a history of breast cancer status after mastectomy, palpable mass, and increased activity on PET/CT may be concerning, although this entity is more likely to be fat necrosis than recurrent tumor.

## 4. Conclusion

Fat necrosis of the breast may be a challenging diagnosis as it has a wide variety of presentations on mammography, ultrasound, CT, PET-CT, and MRI (Table 1). The extent of associated fibrosis, liquefied fat, and calcifications determine the imaging findings of fat necrosis. Mammography is more specific, although ultrasound is still a very important tool in making the diagnosis-increased echogenicity of subcutaneous tissue; in the event of recent trauma, it is the most common presentation and hyperechoic masses are almost always benign. MRI may be helpful in the diagnosis, for example, when internal signal characteristics are identical to those of the adjacent fat and no evidence of enhancement is seen after IV contrast.

## Figures and Tables

**Table 1:** Common imaging features of fat necrosis.

Mammography	(i) Wide spectrum ranging from benign to indeterminate to malignant appearing masses or calcification
	(ii) Visualized masses may be as follows:
	(a) radiolucent with a thin, well-defined capsule
	(b) both radiolucent and dense with encapsulation
	(c) dense and circumscribed mass
	(d) mass with indistinct margins
	(e) mass with spiculated margins

Ultrasound	(i) Sonographic spectrum with two most common appearances:
	(a) mass (anechoic, hypoechoic, isoechoic, or hyperechoic with or without shadowing and enhancement)
	(b) area of increased echogenicity of the subcutaneous tissue with or without small cysts and architectural distortion

CT	(i) Liquefied fat demonstrates low attenuation coefficients (ii) Fibrosis has attenuation similar to fibroglandular tissue or linear densities resembling fibrous bands (iii) Inflammation enhances after contrast injection

PET-CT	(i) Fat necrosis has increased FDG uptake secondary to presence of metabolically active inflammatory cells (ii) It may show intense activity in the setting of TRAM flap reconstruction

MRI	(i) Wide spectrum of appearance depending on amount of inflammatory reaction, liquefied fat, and degree of fibrosis (ii) It may demonstrate enhancement following administration of IV paramagnetic contrast material depending on the intensity of the inflammatory process (iii) Most common appearance are lipid cyst, round or oval mass with hypointense T1-weighted signal on fat saturation images (iv) It is usually isointense to fat elsewhere in the breast (v) “Black hole” sign, marked hypointensity on STIR images when compared with surrounding fat (vi) It may mimic malignancy with thin, thick, irregular or spiculated enhancement
